# Scapular osteochondrolipoma: Imaging features with pathological correlation

**DOI:** 10.3892/ol.2013.1455

**Published:** 2013-07-11

**Authors:** JUN NISHIO, SOSHI IDETA, HIROSHI IWASAKI, MASATOSHI NAITO

**Affiliations:** 1 Department of Orthopaedic Surgery, Faculty of Medicine, Fukuoka University, Fukuoka 814-0180, Japan; 2 Department of Pathology, Faculty of Medicine, Fukuoka University, Fukuoka 814-0180, Japan

**Keywords:** osteochondrolipoma, scapula, magnetic resonance imaging, pathology

## Abstract

Osteochondrolipoma is an extremely rare histological variant of lipoma with osseous and cartilaginous differentiation. The present study reports an unusual case of an osteochondrolipoma occurring in the left scapular region of a 49-year-old male. The physical examination revealed a 3-cm, hard, non-tender and minimally mobile mass. Plain radiography revealed a faintly ossified soft-tissue mass without evidence of bone erosion. Computed tomography (CT) confirmed the presence of a lesion and the normal appearance of the scapula. Magnetic resonance imaging (MRI) showed a well-circumscribed subcutaneous mass with an almost homogeneous high signal intensity on the T1- and T2-weighted sequences. Contrast-enhanced fat-suppressed T1-weighted sequences demonstrated a faint peripheral and septal enhancement of the mass. A marginal excision of the tumor was performed. Histologically, the tumor was predominantly composed of mature adipocytes mixed with thin trabeculae of mature bone. In addition, small amounts of mature hyaline cartilage and osteoid were identified in the periphery of the lesion. Based on these findings, the tumor was diagnosed as an osteochondrolipoma. The patient demonstrated no evidence of local recurrence within six months of follow-up. Although rare, osteochondrolipoma should be considered as a differential diagnosis of a well-defined, calcified/ossified, subcutaneous mass in the scapular region.

## Introduction

Lipoma is the most common mesenchymal neoplasm in humans and may appear in any location of the body (1). The condition has a peak incidence in the fifth to seventh decades of life. Ordinary lipoma usually presents as a soft, slow-growing, painless mass in the subcutaneous tissue or deep soft tissues (2). Cytogenetic analysis has revealed identical translocation, t(3;12)(q27;q13–15), in a subset of lipomas (1). Occasionally, histological subtypes are recognized by a mixture of other mesenchymal elements that form an intrinsic part of the tumor. One example of this is an osteochondrolipoma, which has distinct osseous and cartilaginous components. The etiology of this condition is unclear. The present study reports an unusual example of an osteochondrolipoma arising in the scapular region of a middle-aged male. The differential diagnosis of this tumor is also discussed. Written informed consent for this publication was obtained from the patient.

## Case study

A 49-year-old male was referred to Fukuoka University Hospital with a one-month history of a painless, palpable mass in the left scapular region. There was no history of antecedent trauma. A physical examination revealed a hard, non-tender and minimally mobile mass, measuring ~3.0×3.0 cm. The range of motion of the left shoulder was normal. The neurological and vascular examinations were unremarkable. The patient's medical history was non-contributory.

**Figure 1:**
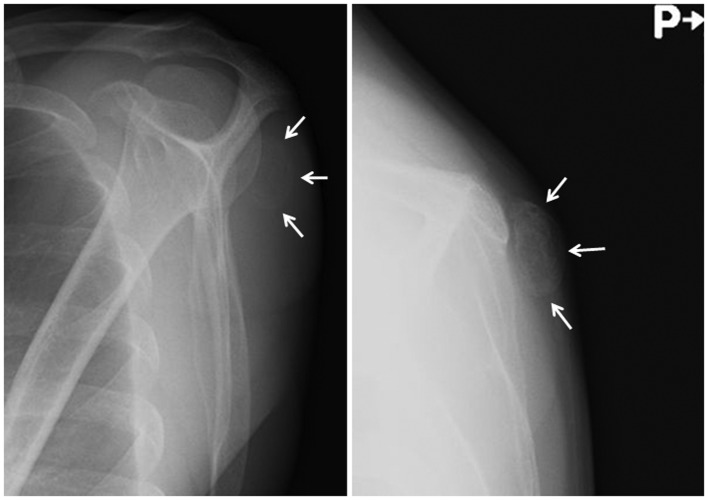
Plain radiographs revealing a faintly ossified soft-tissue mass (arrows), without evidence of bone erosion.

**Figure 2:**
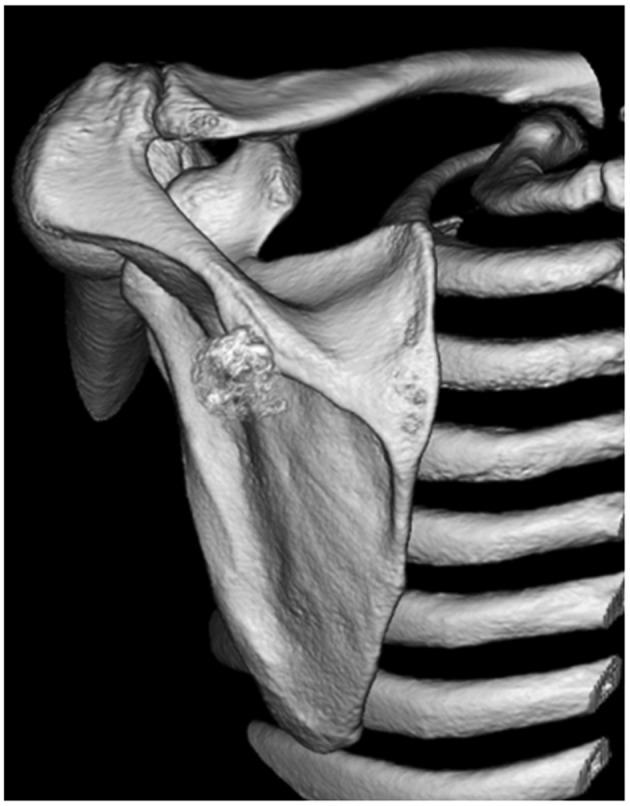
Three-dimensional computed tomography (CT) image showing the presence of a lesion and the normal appearance of the scapula.

**Figure 3:**
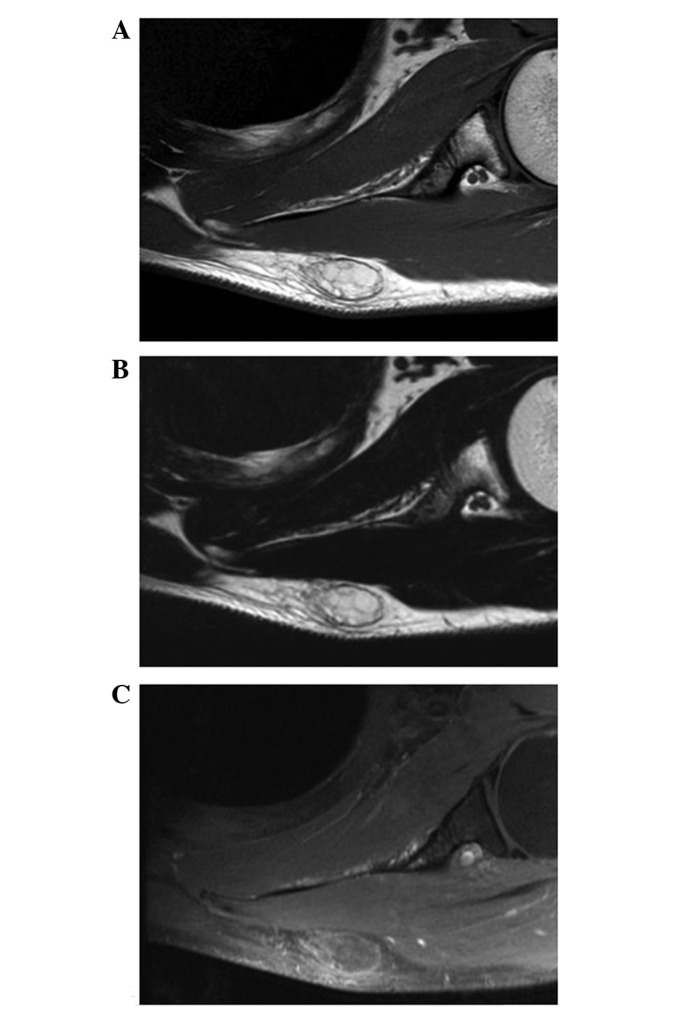
Axial magnetic resonance imaging (MRI) of an osteochondrolipoma in the left scapular region. (A) T1-weighted sequence showing the mass with an almost homogeneous high signal intensity. (B) T2-weighted sequence showing the mass with almost homogeneous high signal intensity. (C) Contrast-enhanced fat-suppressed T1-weighted sequence showing a faint peripheral and septal enhancement of the mass.

Plain radiographs revealed a faintly ossified, soft-tissue mass without evidence of bone erosion (Fig. 1). Computed tomography (CT) demonstrated and confirmed an incompletely ossified shell in the lesion (Fig. 2). Magnetic resonance imaging (MRI) showed a well-circumscribed subcutaneous mass. The mass exhibited an almost homogeneous high signal intensity on the T1- and T2-weighted sequences (Fig. 3A and B). Contrast-enhanced fat-suppressed T1-weighted sequences demonstrated a faint peripheral and septal enhancement of the mass (Fig. 3C). There was no evidence of bone involvement. A diagnosis of a soft-tissue chondroma, extraskeletal osteochondroma or a benign soft-tissue tumor, such as a osteolipoma, was suggested and the lesion was marginally excised.

**Figure 4:**
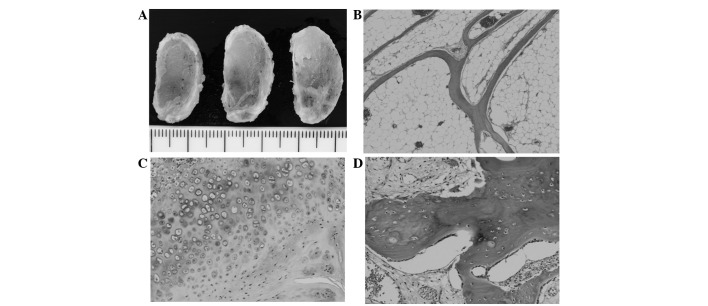
(A) Cut sections of osteochondrolipoma displaying a predominantly yellow, fatty appearance with small white to gray areas. (B) The tumor is composed of mature adipocytes mixed with thin trabeculae of mature bone. (C) Mature hyaline cartilage and (D) bony trabeculae may be observed.

The excised specimen consisted of a well-circumscribed mass with a smooth surface. The cut sections of the mass revealed a predominantly yellow, fatty appearance with small white to gray areas (Fig. 4A). Microscopically, the tumor was predominantly composed of mature adipocytes mixed with thin trabeculae of mature bone (Fig. 4B). In addition, small amounts of mature hyaline cartilage and osteoid were identified in the periphery of the lesion (Fig. 4C and D). Cellular atypia or mitotic figures were not observed. Based on these features, the tumor was diagnosed as an osteochondrolipoma.

The post-operative course was uneventful. At the six-month follow-up appointment, the patient was doing well without evidence of local recurrence.

## Discussion

Osteochondrolipoma is one of the less common histological subtypes of lipoma. The condition may occur at almost any site of the body, notably in the oral cavity (3). Unlike the mass of the present case, osteolipomas tend to be large lesions that have been present for a long period of time (4–6). A marginal excision is curative and local recurrence is rare. An osteochondrolipoma has the same prognosis as a simple lipoma. Recently, a reciprocal translocation t(3;12)(q27;q13–15) has been observed in three cases of osteolipoma (7), supporting the association between osteolipomas and simple lipomas.

The pathogenesis of osteochondrolipomas remains uncertain. Lin *et al*(8) reported that mesenchymal stem cells (MSCs) may be identified in human lipomas and that their characteristics are similar to adipose-derived MSCs. Therefore, the occurrence of osseous and cartilaginous differentiation may be a reflection of the presence of MSCs in certain lipomas. In contrast, certain authors have proposed a hypothesis that ossification/calcification may be caused by repetitive trauma or, possibly, ischemia (4,5,7,9).

CT is of great value in the evaluation of osteochondrolipoma (10). The procedure is useful for documenting the presence of fatty and osseous elements. In the current case, CT clearly demonstrated the presence of surrounding ossification and an association between the tumor and the adjacent bone. MRI is generally considered to be the preferred imaging modality for the evaluation of adipocytic tumors. Simple lipomas have been described as showing homogeneous signal intensities that are identical to subcutaneous fat on all MR pulse sequences, with a complete loss of signal following fat suppression (11). Ossification, calcification and fibrous connective tissue appear as low signal intensity areas on all MR pulse sequences (10,12). In the present case, however, it was difficult to detect and evaluate the peripheral ossifications using MRI.

The differential diagnosis for osteochondrolipoma includes soft-tissue chondromas, extraskeletal osteochondromas, myositis ossificans, ossifying fibromyxoid tumors, chondroid lipomas and well-differentiated liposarcomas.

Soft-tissue chondromas, also known as extraskeletal chondromas, are relatively rare, benign cartilaginous tumors that usually occur in middle-aged adults, with a slight male predominance (13). These chondromas typically present as slow-growing, painless nodules or masses in the hands and feet, particularly in the fingers. MRI usually reveals a low-intermediate signal intensity on T1-weighted sequences and an extremely high signal intensity on T2-weighted sequences (14). Histologically, a soft-tissue chondroma is composed of mature hyaline cartilage with variable amounts of fibrosis or myxoid change. An extraskeletal osteochondroma is a variant of an extraskeletal chondroma that has undergone extensive enchondral ossification (15,16).

Myositis ossificans is a benign, self-limiting condition that predominantly affects active adolescents and young adults, with a slight male predominance. It usually presents as a rapidly-growing, painful mass that expands for six-eight weeks in the extremities, particularly in the anterior thigh. Palin radiographs and CT scans are used to examine this condition and demonstrate the zoning phenomenon, with a peripheral rim of ossification that represents mature bone. MRI usually shows an intermediate signal intensity on T1-weighted sequences and a high signal intensity on T2-weighted sequences (14,17). Histologically, myositis ossificans is characterized by a zonal proliferation of fibroblasts and bone-forming osteoblastic elements.

Ossifying fibromyxoid tumors, first described by Enzinger *et al* in 1989 (18), are rare soft-tissue tumors of uncertain lineage that usually occur in adults, with a male predominance. These tumors typically present as slow-growing, painless, well-circumscribed, subcutaneous masses in the extremities (19). Plain radiographs reveal a non-specific soft-tissue mass with an incomplete rim of ossification. MRI usually shows an intermediate signal intensity on T1-weighted sequences and an intermediate to high signal intensity on T2-weighted sequences (20). Histologically, ossifying fibromyxoid tumors are composed of uniform round, ovoid or spindle-shaped cells that are arranged in nests and cords and deposited in a variably fibromyxoid stroma. A balanced or unbalanced translocation, t(6;12)(p21;q24), appears to be characteristic of an ossifying fibromyxoid tumor (19).

Chondroid lipomas are rare variants of lipomas that usually occur in young and middle-aged adults, with a female predominance. These lipomas typically present as slow-growing, painless masses in the proximal extremities and limb girdles (1). Chondroid lipomas may also exhibit ossification or calcification (21–23). MRI usually reveals a heterogeneous signal intensity on T1-weighted sequences and a variable heterogeneous high signal intensity on T2-weighted sequences (22). Histologically, chondroid lipomas are composed of strands and nests of round cells and mature adipocytes in a myxochondroid matrix. A reciprocal translocation, t(11;16)(q13;p13), resulting in a C11orf95-MKL2 fusion gene, is highly specific for a chondroid lipoma (24).

Well-differentiated liposarcomas are the most common form of liposarcoma that are encountered in late adult life. The condition typically presents as a slow-growing, painless mass in the lower extremities, particularly the thigh. The presence of ossification within a well-differentiated liposarcoma is a rare occurrence (25). MRI usually shows a largely lipomatous mass representing >75% of the lesion and non-adipose components in a thick septa (>2 mm) of irregular aspect or nodular foci (11). Histologically, a well-differentiated liposarcoma predominantly consists of mature adipocytes with a variable number of spindle-shaped cells with hyperchromatic nuclei and multivacuolated lipoblasts. Cytogenetically, a well-differentiated liposarcoma is characterized by the presence of a supernumerary ring and giant marker chromosomes (1).

In summary, the imaging features of an osteochondrolipoma with a clinicopathological correlation have been described. Clinicians should consider osteochondrolipoma as a possible diagnosis for a well-defined, calcified/ossified, subcutaneous mass in the scapular region.
